# Molecular Screening and Single Nucleotide Polymorphism Typing of Molluscum Contagiosum Virus (MCV) from Genital Specimens, between 2012 and 2015

**DOI:** 10.22034/ibj.22.2.129

**Published:** 2018-03

**Authors:** Sedigheh Taghinezhad-S, Amir Hossein Mohseni, Hossein Keyvani, Narges Ghobadi

**Affiliations:** 1 Department of Microbiology, Faculty of Basic Sciences, Science and Research Branch, Islamic Azad University, Tehran, Iran; 2 Research and Development (R&D) Department, Keyvan Virology Specialty Laboratory (KVSL), Tehran, Iran; 3 Department of Virology, Faculty of Medicine, Iran University of Medical Sciences, Tehran, Iran

**Keywords:** Molluscum contagiosum virus, Polymerase chain reaction, Single nucleotide polymorphism

## Abstract

**Background:**

The present study is the first comprehensive report of the Molluscum contagiosum virus (MCV) in Iran based on the molecular technique for differentiation and typing of the MCV1 and MCV2.

**Methods:**

Patients were diagnosed as having tumor-like genital warts less than 5 mm in diameter, and HIV seronegative samples were chosen for this cross-sectional study. TaqMan real-time PCR was used to identify MCV following clinical examination. Typing of the MCV-positive specimens was performed in the SNP A27451G region of *MC021L* gene.

**Results:**

Of 1470 samples, 114 (7.75%) samples were positive for the MCV. From MCV-positive samples, 71.05% sequences were found to be related to the MCV1 and 28.95% to the MCV2.

**Conclusion:**

This assay constitutes a reliable method for identification and typing of the MCV genomic variants that could be valuable for reviewing the pathogenesis, molecular epidemiology, and the natural history of MCV-related situations.

## INTRODUCTION

Molluscum contagiosum is a viral infection of the epidermis that produces multiple umbillicated, tumor-like lesions[1]. This infection has a worldwide incidence, and the typical mode of transmission is via direct human contact, including sexual means or contaminated fomites[2]. Molluscum contagiosum usually affects children but is often associated with immune-suppressed patients with insufficient cellular immunity[3,4]. Molluscum contagiosum virus (MCV) is polymorphic and consists of genomic variants that cluster into two different genetic subtypes, MCV1 and MCV2, each with some variants that have similar patterns of symptoms and occur at similar anatomical sites of the body and have no variant-specific gender preference[5]. There is a substantial conserved genetic structure between MCV1 and MCV2. However, there is a 12-kilo base region specific to MCV1 and a 2-3-kilo base deletion in MCV2[6]. The importance of the alteration between the subtypes is weakly understood, though zonal and age variations in the frequency of the MCV subtypes have previously been reported[7]. MCV1 is the most common infection, while MCV2 may be more widespread in immunocompromised patients[8,9]. Clinically, Molluscum can be confused with skin disorders caused by other pathogens such as herpes simplex virus, varicella zoster virus, and human papillomavirus (HPV), especially in immune-compromised individuals such as HIV patients[10]. MCV can cause a cytopathic effect in some tissue culture lines[11]. The identification of MCV is commonly done clinically. The requirement for laboratory diagnosis MCV is contemplative, since a spontaneous healing is perceived in cases where no underlying immune defect is present. The disease is deliberated as a self-limiting situation, which deserves no more medical attention than an aesthetic nuisance[12]. Consequently, identification of the disease is more valuable when there is an impressive management modality available for the clinician. The genital lesions should be treated in order to break the chain of sexual communication and/or auto-inoculation. When all these features take into account, the practitioner might consider the laboratory diagnosis before starting treatment[13].

PCR-based assays are the best choice for the decisive diagnosis of MCV. An extra advantage of molecular diagnosis is that the results provide data about the subtype of the infecting Molluscum strain[14]. No molecular data have been presented in the literature regarding MCV subtypes prevalent in Iran. Hence, in the present study, we tried to document the possibility of DNA amplification-based assay in the clinical laboratory. This study showed that the first report on the leading Molluscum contagiosum subtypes widespread among the Iranian population.

## MATERIALS AND METHODS

### Study design and population

A total of 1470 Iranian patients (821 females and 649 males aged between 3 and 55) admitted to Firoozgar Hospital in Tehran, Iran between January 2012 to July 2015. Patients who met the inclusion criteria, i.e. patients diagnosed by a general physician or dermatologist as having genital warts caused by HPV or/and MCV and are seronegative for the HIV, were eligible for entrance in our cross-sectional study. Female patients with pre-study Pap smear displaying a high-grade squamous intraepithelial lesion greater than 5 mm in diameter and pregnant or lactating women were excluded from our study. Informed consents were obtained from all participants. Studies included at least one category from three groups. Sample sizes were more than 350 cases for each classification group. According to questionnaires form, 495 cases were classified into immunocompromised patients (group 1), 581 patients had unprotected sexual activities (group 2), and 394 people had the history of swimming in a swimming pool. Some of them were children aged between 5-15 years old who did not cover visible lesions and shared individual instrument such as towel with other friends (group 3). Clinical diagnosis was made in each case according to the morphological and clinical features of the lesions. The number and the distribution of lesions were recorded. Cryotherapy was administered to each lesion during the therapy session as a part of routine anti-Molluscum treatment protocol. After the first visit and treatment, the patients were advised to return for weekly follow-ups in order to assess the success of the treatment regimen. Complete healing was determined as the complete epithelialization and regaining the normal skin appearance at the location of the lesions. The lesions resisting the first treatment, as determined on the fourth visit, were subjected to another cryotherapy. In cases where clinical diagnosis was difficult to reach, lesions were sprayed with ethyl chloride and/or liquid nitrogen. Biopsies from the skin lesions of each patient were cut-off with a sterilized scalpel and placed in 1 ml PBS. Total DNA was extracted from tissue using the NucleoSpin^®^ Tissue Kit (Macherey-Nagel, GmbH and Co, Düren, Germany) according to the manufacturer’s instructions. Validation of the DNA quality was performed with the specific amplification of β-globin gene[15].

### Detection of the MCV in the specimens

TaqMan real-time PCR assay targeting the *MC021L* gene of the MCV was performed. Designed primers and probe by Trama *et al*.[14] were proposed for the assay. In detail, they reported the primers and probe designed according to the conserved region of the MCV1 and MCV2. All primers and probes were purchased from Bioneer Co., Ltd. (Daejeon, Korea). The reactions included 10 µl template DNA (50 ng/µl), 1 µl each primer (300 nM), 0.5 µl probe (200 nM), 12.5 µl Premix Ex Taq™ (Probe qPCR; Takara Bio, Shiga, Japan), and ROX plus (Takara, Japan). The thermal cycles were carried out in an Exicycler™ 96 Real-Time Quantitative Thermal Block (Bioneer, Korea). The temperature profile was 95 °C for 2 min, 95 °C for 20 s, and 60 °C for 45 s for 40 cycles with the FAM and JOE reporter. Furthermore, negative samples for the MCV were examined by specific MY09/MY11 and GP5+/GP6 + primers of HPV[16].

### MCV1 and MCV2 typing

Variable regions of *MC021L* gene (variable between the different types of MCV) were used for molecular typing of MCV. Nucleotide sequences of the target virus (accession numbers HE977596, HE977595, U60315, M63486, HE977606, and M63487) were utilized for primer design by using NCBI Primer BLAST online software (https://www.ncbi.nlm.nih.gov/tools/primer-blast/). The forward and reverse primers sequences were as follows: 5’-CAAGATT GTAGAGACGCTGC-3’ and 5’-GTAGTGCGTGCC GTCCATGT-3’. The forward and reverse primers annealed to the highly conserved regions of 36-55 and 995-1014 of complete coding DNA sequence of *MC021L* gene (accession no. HE977596), respectively. PCR was performed on the MCV-positive genome. The 979-bp fragment was amplified using 1.5 mM MgCl_2_, 0.2 mM dNTP, 1U Taq DNA polymerase, 10× PCR buffer (SinaClon BioScience Co., Iran), 0.5 mM of each primer, and 50 ng MCV genomic DNA . The thermal cycler was run at 95 °C for 1 min, 55 °C for 1 min, 72 °C for 1 min for 40 cycles, and 72 °C for 10 min.

## RESULTS

Of 1470 samples, 114 (7.75%) were positive for the MCV, 1253 (85.24%) were positive for HPV, and 103 (7.0%) were negative for both the HPV and MCV. The resulted PCR products were subjected to direct Sanger dideoxy DNA sequencing with described MC021L typing primers. Sequencing data were first evaluated with BLASTn online software (https://blast.ncbi.nlm.nih.gov/Blast.cgi?PAGE_TYPE=BlastSearch). The results showed 99-100% homology of obtained sequences with *MC021L* gene of the MCV. The nucleotide sequence in the single nucleotide polymorphism A27451G helps distinguish between the MCV1 and MCV2 [14]. This approach was sufficient to discriminate between MCV1 and MCV2 by comparing the expected sequences, but not to identify MCV1 or MCV2 via BLASTn online tool.

**Fig. 1:**
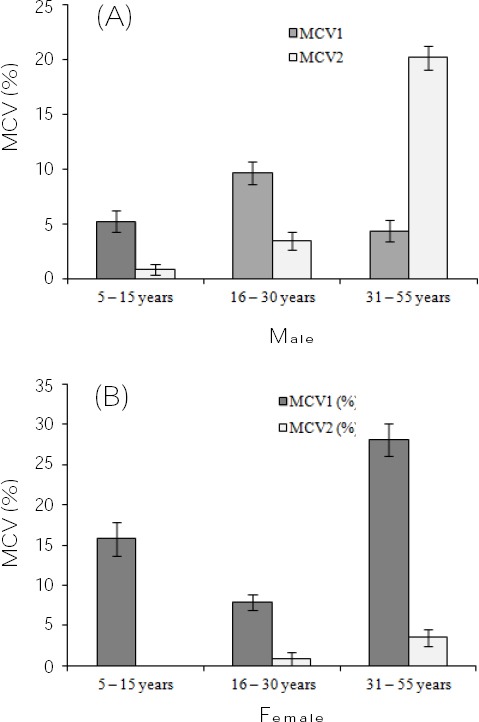
Male (A) and female (B) subtype classification of the MCV positive clinical specimen based on patient gender and age.

**Fig. 2:**
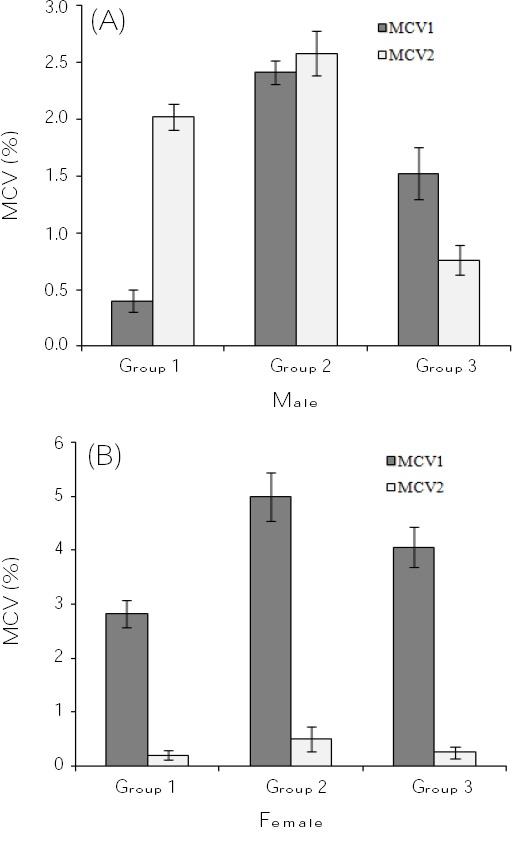
Male (A), and female (B) systematic arranging of the MCV1 and MCV2 on the basis of immunocompromised patients (Group 1), unprotected sexual activities (Group 2), and history of the swimming pool (Group 3).

Finally, the sequence typing of samples was performed with the CLC Sequence viewer (version 6.4; CLC Bio Co., Aarhus, Denmark). The MCV reference sequences were obtained from the GenBank with accessions numbers of NC_001731, MCU60315, and M63486 for the MCV1 and accession number of M63487 for the MCV2. Overall, among 114 positive samples for the MCV, 81 sequences (71.05%) corresponded to MCV1 and 33 sequences (28.95%) matched MCV2, equating to a 2.45:1 MCV1:MCV2 ratio. In accordance with the gender (male or female), the clinical samples were divided into three age groups. The number and distribution of the MCV subtypes according to the ages of the subjects are presented in Figure 1. The maximum percentages of the MCV1 were observed in males of ages 16-30, 5-15, and 31-55 years and females of ages 31-55, 5-15, and 16-30 years, respectively. However, the maximum percentages of the MCV2 in the different ages of males and females were 31-55 years, 16-30 years, and 5-15 years, respectively. Of 114 MCV-positive samples, 27 patients were classified into immunocompromised patients (group 1), 61 patients had unprotected sexual activities (group 2), and 26 people had the history of swimming in a swimming pool (group 3). On the basis of this fact, the highest incidence rate of the MCV1 and MCV2 were related to the patients who had unprotected sexual activities. The results are summarized in Figure 2. Furthermore, the maximum frequency rates of the MCV1 were observed in the female patients, and the greatest rate of the MCV2 were recorded in the male patients. All sequences were deposited in the GenBank with accession numbers of KT289406-KT289519.

## DISCUSSION

Here, we describe a molecular screening and typing of the MCV-specific MC021L PCR assay among Iranian patients, initially established by Trama *et al*.[14]. The results of this study show the possibility of performing dependable and decisive laboratory identification of Molluscum contagiosum with a molecular method during the routine flow of a dermatological practice. The present study, in significance levels, can correspond to different findings of MCV- and HPV-induced mucosal/cutaneous lesions, at least in the anogenital region (particularly in children), where both types of lesions can be found and misdiagnosed. There are incompatible reports regarding the rate of CMV subtypes derived from patients. Saral *et al*.[13] observed MCV1 only in Turkish population. Porter *et al*.[17] reported that the ratio of MCV1 to MCV2 was 3.23:1, and no MCV1 infection was discovered in the patients less than 15 years of age. Equally, in an earlier study, MCV1 was 1.75 times more predominant than MCV2[18]. However, Agromayor and colleagues[19] found an enormous MCV1 infection in a Spanish population with a ratio of 146:1 for MCV1 to MCV2. Yamashita *et al*.[20] assessed 171 Japanese patients using molecular epidemiologic analysis. In their study, MCV1 was commonly detected in children (98%) and adult women (92%), while MCV2 was more frequently isolated from adult men (44%) and from patients with HIV infection (75%). Our results support the previous decision of a high frequency and global distribution of MCV1. In this sense, the high prevalence of the MCV1 in clinical samples occurred in the female patients aged 31-55 years (28.07%), in the men aged 16-30 (9.65%), and in cases who had unprotected sexual behavior (7.4%). In contrast, the predominance subtypes of the MCV2 were in male clinical samples in the age range of 31-55 years (20.17%), as well as in those who had unprotected sexual manners (3.09%) and in immunocompromised patients (5.45%). These results are in agreement with Yamashita *et al*.’s[20] results, suggesting that the possible transmission route of MCV2 is sexual interaction, especially among immunocompromised patients. The disagreements between reported studies might have derived from the methodologies used by different researches. However, the local differences also might be the reason for some of the discrepancies in the infection rates affected by various subtypes.

In Iran, there are limited numbers of publications about the prevalence of the MCV among Iranian population with a narrow range of sampling size. Also, there are no studies that discriminate between the MCV1 and MCV2. However, the current research involved a greater number of lesions samples, providing valuable documents on the epidemiology of MCV infection between our patients. To the best of our knowledge, the present study is the first comprehensive report on MCV infection using molecular technique for differentiation and typing of the MCV1 and MCV2 in different cases (immunocompromised patients, people with unprotected sexual activity, and patients who had a history of swimming in a swimming pool) and ages (5-55 years). However, this study had some restrictions that are its cross-sectional design and the absence of representation of the overall population.

In conclusion, the method can be considered as having sufficient specificity to identify the widest range of MCV variants. Finally, this assay may be valuable for reviewing the pathogenesis, molecular epidemiology, and natural history of MCV-related situations.
